# Managers’ Experience of the Response of the Health System to the Covid‐19 Pandemic for Inpatient Geriatric Care: Lessons About Organisation and Resilience

**DOI:** 10.1002/hpm.3878

**Published:** 2024-11-22

**Authors:** Håkan Uvhagen, Sara Tolf, Karin Solberg Carlsson, John Øvretveit, Maria Flink, Vibeke Sparring

**Affiliations:** ^1^ Research and Development Unit for Older Persons (FOU nu) Stockholm Health Care Services Stockholm Sweden; ^2^ Department of Learning, Informatics, Management and Ethics Medical Management Centre Karolinska Institutet Stockholm Sweden; ^3^ Academic Primary Healthcare Centre Stockholm Health Care Services Stockholm Sweden; ^4^ Division of Family Medicine and Primary Care Department of Neurobiology Care Sciences and Society Karolinska Institutet Huddinge Sweden

**Keywords:** coordination, Covid‐19, crisis management, healthcare, organisational resilience

## Abstract

**Background:**

Effective management of crises is a major challenge for healthcare organisations and their managers. Research suggests that to respond to evolving and unpredictable crises, such as the Covid‐19 pandemic, an organisation needs the capability to continually adapt to the changing situation using relevant knowledge. However, there are few empirical studies using an organisational resilience perspective to understand how a health system responds to this type of crisis. This study aimed to describe managers' perspectives on what influenced the response to the Covid‐19 pandemic in the Region Stockholm healthcare system for older people.

**Methods:**

Data collection was conducted through in‐depth semi‐structured interviews with assistant managers (*n* = 3) and managers of inpatient geriatric services outside of acute care hospitals (*n* = 8), managers of three acute care hospitals (*n* = 3); and the crisis management team for geriatric services (*n* = 3). Data was analysed using qualitative content analysis.

**Results:**

Crisis management of geriatric care in the Stockholm healthcare system during the Covid‐19 pandemic's first 15 months was influenced by a combination of service specific aspects, ‘Internal flexible responses’, collaborative aspects, ‘Coordination within the system’, and governance aspects ‘Adaptive steering’.

**Conclusions:**

This study contributes to empirical knowledge about organisational resilience. Managers' responses are facilitated when the governance allow them more flexibility in their internal responses and enable their cross‐organisational collaboration. A coordinating function across healthcare services is an important enabler in a crisis when the function has well‐established, trustful prior collaborations with the services.

**Summary**

- Empirical data on managers' experience of the health system's response to Covid‐19.
- Importance of organising for evolving crisis instead of time‐limited emergency.
- The significance of collaboration for a resilient adaptive response.
- The necessity of collaboration in the changing post‐covid environment.

## 1 Background

The effective management of crises is a major challenge for healthcare organisations and their managers. If crises are not properly managed, individual health care organisations can be left unable to cope with patient flows, resulting in negatively effects of the entire health system [1]. Many health care organisations have developed capacities to respond to crises [2, 3]: crisis planning has been integrated in strategy processes and plans have been disseminated throughout the organisations related to tactics, training, and media monitoring [4, 5]. However, researchers argue that the organisations have prepared for different crises than what occurred during the Covid‐19 pandemic. Most of the crises management research has been based on events that are *partially* unknown and occurring with some regularity while the Covid‐19 pandemic was a *totally* unknown event that unfolded in unforeseen and inconceivable ways, making it difficult to control, plan and prepare for [3, 6, 7]. As previous crises planning was partly ineffective, and that little help could be found in experience or guidance from government agencies [8], the Covid‐19 pandemic demanded different kind of capacities [2].

Unlike the partially unknown events, there is no single model of response for the totally unknown events, but researchers have identified several important features of the latter. An important capacity in the totally unknown events is to improvise decision‐making depending on what turns up and to flexibly scale and mobilise resources across different levels of the organisation [8, 9]. Another important capacity is to take risks by balancing bureaucratic and static models with flexibility [10, 11, 12, 13]. The ability of the health system and the managers to respond quickly and effectively call for a high degree of flexibility provided by organisational structure [14] including the capacity to quickly reconfigure existing processes in response to sudden changes in demand [15]. In addition to flexibility, health systems ability to effectively respond to crises also depends on the communication and collaboration within and between all levels of the system [16]. At a higher level of organisation, communication and collaboration between services and welfare sectors involved in emergencies is especially necessary [17]. Such collaboration requires coordination structures and systems, as emphasised in emergency management literature and practical guidance [18]. In all, in times of high uncertainty, managers need to develop capacities to enable them to effectively cope with unexpected events [19]. Managers also need the adaptive capacity to absorb shocks within existing resources while keeping essential functions as before, and to adapt by adjusting functions and resources [20].

The concept of organisational resilience has been suggested as a way to understand how an organisation impacted by an unexpected event, recovers and responds [21, 22] and has increased in popularity with the increase of a growing uncertain and unpredictable environment. Many organisations are designed to operate in a stable, predictable and routine environment and thus get vulnerable in a more volatile world [23]. Duchek [19] describes resilient organisations as having the ‘capabilities that enables them to adapt, integrate, and reconfigure internal and external resources and competences to match the requirements of changing conditions.’ It is also emphasised that organisational resilience takes on a multisystem perspective and require action at all levels of the system [22].

Our review of literature discovered some research about responses to time limited crises or partially unknown events. However, at the time of the study we found no empirical data about managers' experiences of and adjustment to an ongoing crisis such as a pandemic involving an entirely new and unknown virus. Though encouraged by researchers, few studies have provided an empirical perspective on organisational resilience or studies of managers' real world coping strategies of crises [22, 24, 25].

**FIGURE 1:**
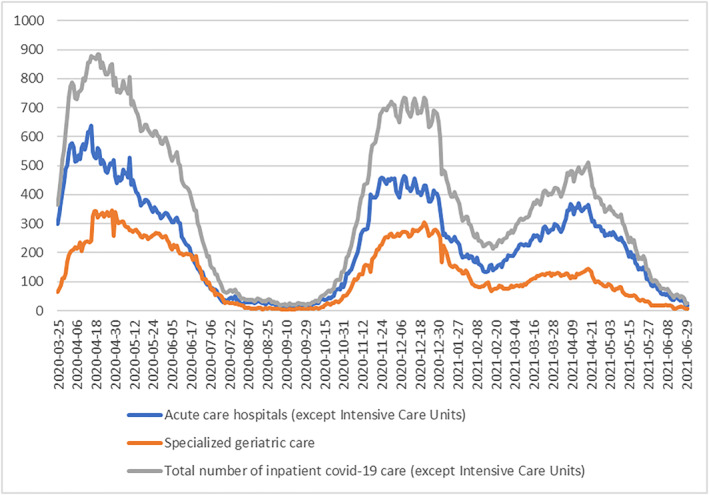
Public data concerning inpatient care in the Region Stockholm during the first 15 months (25 March 2020–2030 June 2021) of the Covid‐19 pandemic, by Acute care hospitals (except Intensive Care Units), specialised geriatric care, and total number of inpatient Covid‐19 care (except Intensive Care Units).

This study was conducted in Region Stockholm, the largest healthcare region in Sweden with approximately 2.3 million residents. Public data about the delivery of inpatient Covid‐19 care in Region Stockholm between March 2020 and June 2021 shows that the inpatient geriatric services provided between 12% and 58% (mean 33%) of the region's total inpatient Covid‐19 care (Figure 1) [26].

The management of the Covid‐19 pandemic in Region Stockholm is an example of how organisations work and cooperate in new ways in the event of a type of crisis than those they are usually trained for. Our study aimed to describe and understand managers' perspectives on what influenced the response to the Covid‐19 pandemic in the Region Stockholm healthcare system for older people.

### 1.1 Inpatient Geriatric Services for Older People in Region Stockholm, Sweden

The regional healthcare system in Stockholm, Sweden, includes contracted private and public‐owned service providers [27]. Healthcare in Region Stockholm is mainly tax‐financed and organised in a purchaser‐provider model. This means that the service providers are regulated and funded by service contracts by the regional purchasing office. Inpatient geriatric services (hereafter named geriatric services) in Region Stockholm are organised differently to those internationally. There are a distinct set of geriatric service facilities outside the acute care hospitals, 11 in total at the time of this study, with sub‐specialist in‐ and outpatient services operating from these facilities. Nine of the geriatric services are privately operated and owned but are publicly funded and contracted and independent from the acute/emergency hospitals. Staff include geriatricians, registered nurses, and allied health professionals trained for geriatric patients, mostly working in multidisciplinary teams [28].

Region Stockholm's contracts with the geriatric services stipulate a geographical responsibility for each, but the patient has the right to choose another service if care beds are available elsewhere. Specialised geriatric care is normally provided to people over 65 years of age (mean age 83 years in 2019), for assessment, treatment, care planning and short‐term rehabilitation after emergency hospital inpatient care for medical or surgical conditions. Patients receiving specialised geriatric care are often frail, have several medical conditions, and undergo different treatment procedures often in parallel [28, 29]. In 2019, the geriatric services provided 37,475 care episodes and the average length of stay was approximately 8 days [30]. The patients are most frequently admitted from the acute care hospitals (approximately 80%) or directly from their ordinary homes. About three out of four patients are discharged to their ordinary homes [29, 31]. Social home care services and nursing homes for older people are the responsibility of the 26 municipalities within the region [31].

## 2 Methods

### 2.1 Design

An exploratory study design, drawing on data from qualitative interviews.

#### 2.1.1 Setting: Organisation of Crisis Management During the Covid‐19 Pandemic in Region Stockholm

**FIGURE 2:**
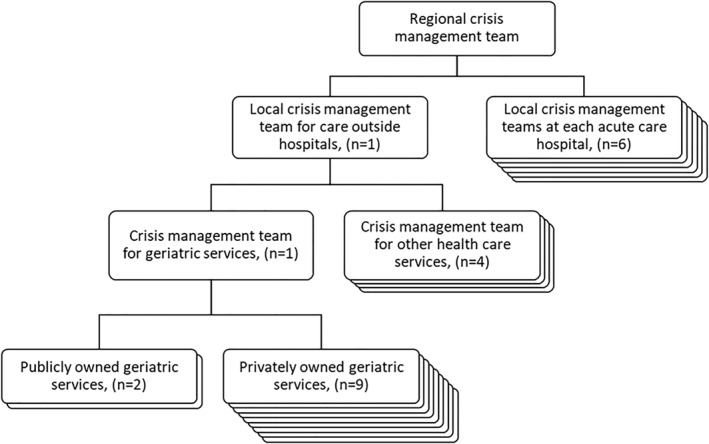
Organisation of Region Stockholm's crisis management.

The first positive case of Covid‐19 in Region Stockholm was confirmed by the end of February 2020. Approximately 3 weeks earlier, the government of Region Stockholm activated the *regional* crisis management team [14, 32]. In case of ‘extraordinary events’, the regional crisis management team is responsible for regional coordination of healthcare and collaborations with other authorities and organisations [32]. Reporting to the *regional* crisis management team, *local* crisis management teams for specific parts of the healthcare system were activated in the region (see Figure 2). The public owned healthcare service provider Stockholm Health Care Services was delegated to execute a local crisis management team on the 28th February 2020, specifically targeting all public‐owned and private healthcare services outside the acute care hospitals and with the mandate to coordinate services, re‐distribute resources, and re‐locate patients [14, 26]. The local crisis management was organised into smaller crisis teams with responsibility for different parts of the healthcare system outside the acute care hospitals, for example primary care, psychiatric care, and geriatric care. The crisis management team for geriatric services outside the acute care hospitals was led by four persons: a head crisis manager and three deputy crisis managers, all with long clinical experience from specialised geriatric care in the region.

#### 2.1.2 Participants and Data Collection

We invited the total population of managers of the geriatric services outside the acute care hospitals (*n* = 11), managers of the three largest acute care hospitals (representing approximately 75% of the acute care beds), and the complete crisis management team for geriatric services (*n* = 4) to participate in the study. Individual interviews were conducted with 12 participants: managers of geriatric services outside the acute care hospitals at two public and four private service providers (*n* = 6); managers from three acute care hospitals (*n* = 3); and the crisis management team for geriatric services (*n* = 3). Two interviews were conducted with both managers of geriatric services and their assistant managers from two private service providers (*n* = 5). In total, 17 managers were interviewed. By letting the notion of *information power* [33] guide our determination, we considered the information to be sufficient to substantiate our analysis.

Participants were informed about the study through email and, if they were interested in participating, asked to schedule an interview. In the email, the research project and its objectives were described, and it was stated that the participants had the right to withdraw from the study at any time. After oral information had been given, audio‐recorded informed consent to participate in the study was obtained from all participants. The interviews were conducted online in June 2021 using a video call application. The interviews lasted between 26 and 67 min (mean 44 min), were audio recorded on a digital recorder and transcribed in verbatim. Separate, semi‐structured interview guides were used for each manager group. Prior to the interviews, a pilot interview was conducted by J.Ø. The questions were open‐ended with an overall focus on the managers' perspectives on the conducted changes to regular services, collaborations between services, and what facilitated or hindered these changes. The data collection was conducted by the authors H.U., K.S.C., M.F. and V.S. The authors regularly met during the interview period to discuss the interviews and the data collection.

### 2.2 Data Analysis

The interview data were subject to conventional content analysis [34]. This approach allowed us to inductively analyse the data to gather a condensed description of the managers' response. In the first step, the coding procedure was outlined whereafter the authors H.U., K.S.C., M.F. and V.S. individually coded one transcribed interview each. The authors read their interview word by word and identified meaning units that contained information related to the study aim. The meaning units were labelled with a code. The coding was conducted in Word to allow sharing of the coded interviews between the authors. The authors discussed the coded interviews to ensure a similar coding procedure in the further analysis. The codes were kept on a manifest level, close to the participants' original descriptions, and compared to the meaning units several times before further analysis. In the following step, the interviews were divided between the four authors for analysis, resulting in approximately 800 codes. The authors together sorted the codes based on relations and similarities between the codes into meaningful clusters. This resulted in 30 sub‐categories. In the last step, the sub‐categories were sorted into three categories (see example Table 1). The clustering of codes into sub‐categories and sub‐categories into categories was carried out by all analysing researchers together, where the codes, sub‐categories and categories were discussed until consensus was reached. After the authors agreed on categories, quotes that represented different managers were selected. All participating managers of the geriatric services outside the acute care hospitals as well as the crisis management team for geriatric services were invited to a 45‐min digital respondent validation activity [35]. The results were presented on a category level to four participants followed by a discussion to verify accuracy and ensure it resonates with their experiences. The participants raised no concerns or suggestions for changes.

**TABLE 1:** Example of the analysis.

Meaning unit	Code	Sub‐category	Category
All of a sudden, we just started to do this thing together and it felt like it was like that throughout the entire [care] chain. And it was absolutely fantastic, that it wasn't like we and them, or that ‘this will be a burden on us’, or ‘will we lose revenue?.’ Everybody just threw those thoughts out the window and said ‘let's just deal with this pandemic’. And then I believe… If you have those prerequisites, then… Then you'll succeed.	If the entire care chain works together, we will succeed	The crisis highlighted collaboration	Coordination within the system

In the following result section, quotations that exemplify the identified categories are used to illustrate the findings. Each quote is identified by a letter that designates a specific interviewee. In the transcript excerpts, the symbol/…/indicates an omission, and [] indicates an addition to enhance readability or secure anonymity of the participants. All presented quotes have been translated from Swedish.

## 3 Results

**FIGURE 3:**
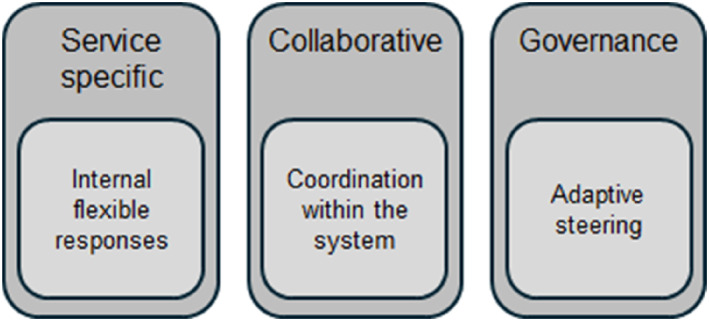
The categories and their related aspects.

The analysis shows that the crisis management of geriatric care in the Region Stockholm healthcare system during the Covid‐19 pandemic's first 15 months was characterised by a combination of three main categories, that influenced the management. The categories relate to service‐specific aspects, ‘Internal flexible responses’, collaborative aspects, ‘Coordination within the system’, and governance providing ‘Adaptive steering’ (Figure 3).

### 3.1 Internal Flexible Responses

The interviewees described that, in early March 2020, all service managers were informed about the new crisis management organisation and that their organisations must prepare for caring for older people infected by Covid‐19. The following day, they reported that the service managers responded by gathering their management groups and by brainstorming ideas for management of a clinical response for patients with new and acute needs. The managers revised their internal structures and processes including re‐organisation of internal management team, for example by distributing new responsibilities for subordinate managers and rearranging meetings. Managers reported rearranging care settings such as changing quadrable patient rooms to single rooms and arranging separate wards for patients with Covid‐19 to avoid contagion between individuals. Clinical level managers reported organising the wards for patients with Covid‐19 with staff qualified for and acquainted with working with highly contagious patients and infection control. The interviewees also described that their ability to respond was aided by previous simulation training for different types of crises.

We pretty quickly had to redistribute our patient beds because we realised that if they [the beds] were situated in quadruple rooms, we would not be able to utilise them. We made more single rooms and double rooms out of larger rooms to be able to keep a decent distance between patients.

(C)

During the first months of the pandemic, nearly all managers in the sample described struggling to stay up to date with the large amount of information from both formal sources, such as the healthcare region, and from informal sources, such as social media groups and online lectures from other countries. New directives, recommendations, and reports were initially received daily. Despite the large amount of information, there was a lack of knowledge about Covid‐19, which made it difficult to anticipate what was about to happen. The managers described difficulties in making quick decisions and communicating them, while acknowledging the unpredictability of the situation and having to be ready to reconsider and change previous decisions. This required new ways to spread information quickly and broadly in their own organisations to maximise staff understanding, yet still reminding staff that this was provisional and subject to change. An example of a solution reported to handle the large amount of information received were daily telephone conferences with all lower‐level managers within the organisation to ensure that all had heard the same information.

Many participants described a work situation that was almost unbearable, which included coping with the massive information flow, added tasks, feelings of heavy responsibility to take care of their own patients, and limited access to personal protective equipment. Managers described that, in some cases, the geriatric services had personal protective equipment only to cover the next few hours, without knowledge about availability the next day. To manage these practical and emotional challenges, managers reported trying different ways to acquire personal protective equipment, and, in collaboration with the personnel, they worked out innovations such as making their own personal protective equipment. The interviewees highlighted the importance of always having a manager physically present for quick responses to staff's questions, which was important for creating a sense of safety for staff.

Managers reported staff shortages due to sick leave related to Covid‐19 symptoms during the first months of the pandemic; towards the later phases of the pandemic, sick leave was increasingly associated with staff fatigue. During all phases, Covid‐19 required more advanced and resource‐intensive care. To meet these staff challenges, task‐shifting and task‐sharing was implemented: kitchen staff and allied health professionals were given introductions to caring tasks, kitchen staff were replaced with restaurant staff, and personnel in orthopaedic services were reallocated to work in the geriatric services. Staff classified as being within a risk group were reallocated to work with patients other than those with Covid‐19.

We moved allied health professional staff, gave them rapid training in basic care and assigned them to the wards. Those working in the kitchens who had experience in a care environment, were assigned to the wards. And then we took exempt chefs from the restaurant business and manned our kitchens.

(D)

### 3.2 Coordination Within the System

The interviewees reported that they recognised more than before that the whole system needed to work ‘as one’, instead of in ‘silos’ to give adequate care for all patients. This led to an increased understanding of the work and responsibilities of different services, as well as a feeling of common purpose, and a new‐found respect and knowledge of collaborating partners.

It has given us quite a lot of knowledge about each other's organisations and challenges. I mean, it's easy to have an opinion about a lot of things, about things you don't know about, and what others should do. But the thing is, you must look at yourself and understand others' challenges. /…/ So it has taught me a lot about other healthcare services that I didn't know much about.

(E)

Coordination between geriatric services, acute care hospitals, primary care, and municipalities was reported to improve over time during the pandemic and was strengthened by perceptions of a common responsibility and goal that could only be achieved by working together. One example given was that the local coordinators at each geriatric service and coordinating functions within the municipalities intensified their internal collaboration to ensure safe admission and discharge of the patients from the geriatric services. Existing collaborations, forums for discussions, and relationships with each other prior to the crisis were described as important for their ability to respond quickly.

The interviewees reported that, in order to avoid the chaos seen in hospitals abroad in the early phases, the geriatrics services helped reduce the demands on the acute care hospitals in the region. Similarly, the geriatric services were aided by others to ensure efficient transfers to discharge patients and release care beds. The interviewees described how the municipalities and primary care services made several adjustments to transfer patients, such as increasing the number of staff during weekends and out‐of‐office hours, and adding interim beds. This was reported to have played an important role in facilitating the patient flows from the geriatric services, thus freeing up care beds both there and in the acute care hospitals.

And if I think about other actors like the municipality and primary care, then the municipality… Above all, the municipality has been fantastic, I must say.

(B)

Many interviewees emphasised the important coordinating function of the geriatric crisis management team. As this team worked across the system—having contact with acute care hospitals, other healthcare outside the acute care hospitals, municipalities, and the purchaser organisation—they solved collaborative issues and contributed to knowledge exchange. This team had daily contact with the managers of the geriatric services to check what assistance or information they needed. They established a shared digital information and peer support group for the managers at the beginning of the pandemic, with daily updates, which facilitated the sharing of knowledge and experiences between professionals.

### 3.3 Adaptive Steering

Interviewees highlighted the significance of both centralisation and decentralisation of different decisions for managing the crisis. The centralised steering by the geriatric crisis management team was described as central to maintain a system‐wide perspective when needed, for example regarding patient transfers. Instructions and working rules to ensure an optimal system‐wide performance placed limits to the autonomy of lower‐level managers and staff. However, a key aspect for success was delegation by centralised steering group close to where care was provided, which increased the managers' decision space, allowing flexible processes and coordination.

An in‐depth understanding of the health system was reported by both managers and the crisis management team as a key factor for an effective response. Many managers perceived that the steering by the geriatric crisis management team was supportive and related this to the team's personal experience of geriatric care and in‐depth knowledge of medical care and the healthcare system at all levels. The interviewees emphasised that this knowledge enabled the team to a deeper understanding of the consequences of decisions for practice, organisations, and patients. The crisis management team, on their hand, perceived that the managers' understanding of their staff's situation enabled their response.

I think they [the managers of geriatric services] succeeded through a “management close to the clinic”. They understood care, understood the challenges. And could give support in that.

(A)

The interviewees acknowledged that the temporarily reduced bureaucracy enabled the managing of more seamless care transitions for patients. For example, at the beginning of the pandemic, the services reduced the threshold for admission to increase patient accessibility and made care transitions between services easier and more flexible. Previous criteria to only admit patients within one's own geographic area were removed, which opened for better usage of available beds. The initial central decision to suspend routine, non‐essential tasks, such as documentation for some registers, risk assessments and other quality projects allowed freeing up time for the services to concentrate on patient care.

## 4 Discussion

This study reports on the perspectives of managers on the health systems' response to the Covid‐19 pandemic. Three aspects, that influenced the management, were the service‐related ‘Internal flexible responses’, collaborative‐related ‘Coordination within the system’, and governance‐related ‘Adaptive steering’. As the Covid‐19 pandemic was a complex and evolving crisis, i.e., being multi‐factorial and multi‐scale in cause and effects in a multi‐level system [20], the findings of this study can be considered as a description of the resilience of one health system and contribute to the understanding of health organisation's systems capacity to absorb shocks with the resources at hand. The pandemic provided an opportunity for our team of researchers to study a response to the pandemic from the managers' perspective.

The managers' internal response included their capacity to simultaneously and quickly respond to many different types of challenges, without compromising system performance. Their responses included e.g., reorganising the management team, rearranging care settings, and staying up to date with a large amount of constantly changing instructions and policies and uncertain information. Also reported was the work of finding ways to communicate the information to staff and being physically present to ensure their staff felt safe. Together this required a high level of flexibility and placed a high workload and decision‐making demand on the managers. Our findings correspond to Gröschke et al.'s study of leadership characteristics contributing to resilient organisations. This leadership has been characterised by visibility, availability, transparency, and inclusive decision making [36]. Such capacity in leadership has been shown to play a crucial role for an organisation's resilience in acute shocks, such as the Covid‐19 pandemic [25]. In addition, systems are more flexible when decisions are made close to the operational level. Therefore, decentralised decision‐making, participation and accountabilities to lower levels are important features of adaption in crises [24]. In this study, the managers applied a decentralised decision‐making leadership as they brainstormed ideas with their co‐leaders about how to proceed, and innovated together with the personnel. This inclusive approach differs from that reported by some other studies in which managers applied a ‘top‐down’ management approach [37].

The managers reported that the specialised geriatric services had to quickly adapt their work practice and organisation of work due to the continuously changing situation, not least because older people were most at risk of serious illness or death. For example, task‐shifting was used for tasks requiring non‐medical skills, and task‐sharing for tasks requiring medical knowledge; a method described as important to maintain safety [38], and applied in other countries during the pandemic [39, 40]. Our results discovered the importance of capacity and competence in operational work, but also the significance of a context that allowed flexibility to respond to sudden changes in demands, staffing and regulations. These findings correspond to previous research [22, 41], emphasising the importance of flexible management and reconfiguration ability to respond to changing governance and demands to unpredictable and turbulent events.

Coordination within the system, i.e., the capability to collaborate with other parts of the system and arrangements to enable this, appeared from this study to be an important feature in managing the Covid‐19 pandemic crisis. At all levels, collaboration was enabled by staff having a shared and critical purpose: to minimise infection and to diagnose and treat both Covid‐19 illness and other illness. At the clinical level, as well as in management teams, teamwork was reported to be essential, and this has been highlighted as a key factor in resilient organisations [42]. Others have also highlighted the importance of a comprehensive systems perspective and the capacity to link different parts of the system, where the success of the separate services not only depends on their individual performance and contribution but also on their ability to collaborate with each other [16]. Our study found that the coordinating function of the crisis management team that focused on building relationships and cooperating with all organisations on common pressing issues was crucial to achieve system effectiveness. The geriatric crisis management team had previous experience and working relationships with managers across the healthcare system; this enabled them to create spaces for coordination across the system and have daily contacts with managers [16]. As reported by Koenig and Schultz [17], coordination, in our study performed by the local crisis management team, appeared to involve different competencies. On the one hand, management support to separate services benefits from an understanding of the specific context and content of care. On the other hand, coordination at system level is based on a broader management understanding of the system and how the different services connect and interact. In addition, trust between the coordinating crisis management teams and the services coordinated also appear to have been an important enabler during the crisis.

Prior collaborations and trustful relations between health and social care services before Covid‐19 was also reported as facilitating a collective response to the pandemic and collaboration when needed. Our findings complement those of Barasa, Mbau, and Gilson [25], who suggests that organisations need to plan and prepare for crises by emphasising that organisations will gain from investing the time and efforts to create structures for collaboration and build trustful relations with partners in both health and social care in a future crisis. We understood this to mean that collaborations can be formalised and implemented during crisis events, but that there is a potential risk of not having time, nor the ability to achieve this if not previously developed, because this work competes with patient‐related, acute activities.

During the pandemic, the regional governance shifted towards a centralised steering with coordination delegated to the crisis management team and a simultaneous decentralisation of decisions delegated to specialised geriatric services. The governance also reduced the bureaucracy which facilitated work processes and collaboration. This corresponds with Hartney et al.’s [43] conclusion that collective agreements and bureaucratic hierarchical management systems ‘slow down innovation and change’ and their argument that, in crises, when quick problem solving is needed it is necessary to let managers use their professional judgement. Our findings of this indicate that the pandemic created opportunities for managers to exercise their professional judgement and decision‐making for swift problem solving, or to take the initiative to do so even if some rules and policies hindered this. Although there is a need for follow‐up and steering at different levels, this raises the question of whether some of the bureaucracy during normal circumstances may be substituted for a more flexible trust‐based steering during crises, and indeed, whether it is now necessary during a turbulent post‐covid environment for healthcare.

### 4.1 Global Lessons From the Swedish Model

The findings of this study share both similarities and differences compared to other countries. Similar findings about highly centralised and inadequate governance early in the pandemic was also reported in many European healthcare systems, resulting in confusing information, unclear guidelines, lack of equipment and staff, and lack of coordination between healthcare services [44]. A distinctive finding from our study was a specific type of decentralisation: initially there was strong formal central detailed prescription with the emergency regulations, which was then adjusted to allow for significant autonomy to units, within broad parameters defined centrally, which we termed ‘adjuster appropriate decentralisation’. The ‘appropriate’ referred to the capability and experience of managers with exercising their autonomy, which had been developed over the years in this healthcare system [45]. Other studies reported both formal decentralisation [39] (Japan) and units informally ignoring the rules but no capacity to adapt appropriately and this combination (noted before the pandemic by Sujan, Spurgeon, and Cooke [46]).

We found that the capacity to link different parts of the system was important: both linking vertically and horizontally. An example of a vertical link was within the geriatric care, were significant task‐shifting was conducted, which also has been reported in Japan [39, 40]. Horizontal links were found in the geriatric crisis management teams, who had previous experience and working relationships with managers across the healthcare system, which enabled a daily contact with managers [25]. Whilst other general studies and theories reported these activities, e.g., the importance of collaborations and trustful relations between health and social care services before Covid‐19 was reported in general in other studies [25], none reported empirical findings related to geriatrics (e.g., Koenig and Schultz [17]). Overall, the importance of a resilience capacity to enable a continuing adaptive response was also noted by Mannion et al. [47] for the National Health Services as a lesson from responses to Covid‐19.

### 4.2 Policy Implications

The risk and frequency of shocks to the health care system will probably increase. It is therefore essential for policymakers to be able to prepare for, steer and manage their health systems through various shocks. This study highlights that pre‐existing collaboration and trust between different parts of the healthcare system facilitated a coordinated response, and why policies should encourage the development of inter‐organisational partnerships and joint exercises to prepare for future crises. Investing in collaborative structures for relationship‐building and increased system‐understanding before a crisis strikes could facilitate more seamless coordination during emergencies. Health system planners and policymakers should incentivise integrated healthcare planning and coordination between acute care hospitals, geriatric services, municipalities, and primary care. A formalised structure for collaboration across sectors, such as crisis management teams, might foster smoother transitions and communication. A key finding is that reduced bureaucracy and adaptive steering enabled more efficient crisis management. Policymakers should consider creating flexible governance models that allow temporary suspension of routine administrative tasks and quality assurance processes during crises. This can free up resources for direct patient care and improve the system's overall responsiveness. Also, the governance measures adopted during the crisis, such as reduced bureaucracy, may be relevant in the post‐pandemic healthcare environment. Policymakers should assess which regulatory flexibilities can be maintained to improve efficiency in the long term without compromising accountability or safety. In summary, policymakers would benefit from developing frameworks for organisational resilience, emphasising leadership capacity, decentralised decision‐making, and system‐wide collaboration. Healthcare systems need the structural capacity to absorb shocks and continue functioning during crises, as seen with the Stockholm geriatric care response.

### 4.3 Methodological Considerations

Although all managers of the geriatric services outside the acute care hospitals and managers of the three largest acute care hospitals in the region were included, they represent only a specific part the healthcare sector. This is related to the study's aim to describe the response in the setting of geriatric care, but the results clearly showed how changes in one service impacted other services, such as municipalities or primary care. The experience of a wider group of stakeholders would have contributed to understanding the interdependencies and the adequacy of the coordination structure to enable adjustment between services. However, generalisability pertains to the extent to which research findings can be applied to other contexts or populations. Considerations of generalisability are often suggested rather than explicitly claimed. Ultimately, it is the responsibility of the reader to determine the applicability of the findings to other contexts [48]. To facilitate this assessment, we have tried to provide comprehensive descriptions of the context, data collection methods, data analysis procedures, and illustrative quotations, all aimed at enabling readers to critically evaluate the generalisability of the findings to their specific contexts.

One strength of the study is that the interviews and analysis were conducted in collaboration between four authors with thorough discussions of the analysis process and the results. Another strength is the respondent validation with four of the subjects that gave us an opportunity to further reflect on the analysis and results.

## 5 Conclusion

This study contributes to empirical knowledge about organisational resilience with findings about the capacity to continuously adapt to a changing situation. This capability may be necessary in the new turbulent post‐covid environment for healthcare. The structures that enabled managers' response was related to internal, collaborative and governance factors. Managers' responses were facilitated when the governance allow them more flexibility in their internal responses and enable their cross‐organisational collaboration. A coordinating function across healthcare services is an important enabler in a crisis when the function has well‐established, trustful prior collaborations with the services.

## Author Contributions

All authors participated in designing the study. Prior to the 14 interviews, a pilot interview was conducted by J.Ø., H.U., M.F., K.S.C. and V.S. collected the data, conducted the analyses, and drafted the manuscript. All authors read, contributed to, and approved the final manuscript.

## Ethics Statement

The Regional Ethics Committee in Stockholm has granted ethical approval for the study (ref No. 2020‐01521, and amendments ref Nos. 2020‐02928 and 2021‐02242). Participation was based on informed consent and participants were prior to the interview given written information about the project. All participants have been given the opportunity to go through the presented results to validate information and comment on any misunderstandings.

## Conflicts of Interest

All authors were at the time of the study employed by the Stockholm Health Care Services. H.U. was part of the crisis management team for geriatric care.

## Data Availability

The data that support the findings of this study are available on request from the corresponding author. The data are not publicly available due to privacy or ethical restrictions.
